# Low Reported Adherence to the 2019 American Diabetes Association Nutrition Recommendations among Patients with Type 2 Diabetes Mellitus, Indicating the Need for Improved Nutrition Education and Diet Care

**DOI:** 10.3390/nu12113516

**Published:** 2020-11-15

**Authors:** Savvas Katsaridis, Maria G. Grammatikopoulou, Konstantinos Gkiouras, Christos Tzimos, Stefanos T. Papageorgiou, Anastasia G. Markaki, Triada Exiara, Dimitrios G. Goulis, Theodora Papamitsou

**Affiliations:** 1 Department of Nutritional Sciences & Dietetics, Faculty of Health Sciences, Alexander Campus, International Hellenic University, Sindos, GR-57400 Thessaloniki, Greece; savvaskatsaridis@gmail.com (S.K.); mariagram@auth.gr (M.G.G.); 2 Unit of Reproductive Endocrinology, 1st Department of Obstetrics and Gynecology, Medical School, Aristotle University of Thessaloniki, Papageorgiou General Hospital, GR-56429 Thessaloniki, Greece; 3 Laboratory of Clinical Pharmacology, Faculty of Health Sciences, Medical School, Aristotle University of Thessaloniki, GR-54124 Thessaloniki, Greece; kostasgkiouras@hotmail.com; 4 Northern Greece Statistics Directorate, Hellenic Statistical Authority, 218 Delfon Str, GR-54646 Thessaloniki, Greece; ctzimos@gmail.com; 5 Faculty of Health Sciences, Medical School, Aristotle University of Thessaloniki, GR-54124 Thessaloniki, Greece; papagesn@auth.gr; 6 Department of Nutrition and Dietetics, Faculty of Health Sciences, Hellenic Mediterranean University, PO Box 8556, Trypitos, GR-72300 Sitia, Greece; markakin@hotmail.com; 7 Department of Internal Medicine, Sismanoglio General Hospital, 45 Sismanogliou Str, GR-69133 Komotini, Greece; texiara@otenet.gr; 8 Laboratory of Histology and Embryology, Medical School, Aristotle University of Thessaloniki, GR-54124 Thessaloniki, Greece

**Keywords:** diabetes guidelines, nutrition guidelines, dietary supplements, lifestyle medicine, carbohydrate counting, diabetes education, HbA_1c_, medical nutrition therapy, glucose-lowering drugs, health equity

## Abstract

Patient adherence to guidelines is important for improved outcomes and prognosis. Nevertheless, many patients with type 2 diabetes mellitus (T2DM) do not comply with the recommendations regarding medication, physical activity, diet or self-care. The present cross-sectional study aimed to assess the level of adherence to the dietary recommendations issued by the American Diabetes Association (ADA) among patients with T2DM in Komotini, Greece. A total of 162 adults with T2DM (64.7 ± 10.6 years old), of which 41.4% were men, were recruited from the Sismanoglio Hospital and participated in the study. The level of adherence to individual recommendations issued by the ADA was assessed using yes/no questions. The overall adherence rate to the guidelines was low (41.2%). According to the multivariable analysis, age and medication therapy were identified as contributors to the compliance rate. No differences were noted in the total compliance rate between patients of different religious denominations (Muslims/Christians). Patients on oral antidiabetic agents (OAA) were more adherent compared with those on insulin therapy. A mere 3.7% of the participants had received nutrition education by a registered dietitian, 9.9% were following an individualized diet plan to improve glycemia, and 3.1% had set specific energy goals to reduce body weight. These findings are indicative of the need for the delivery of improved nutrition education.

## 1. Introduction

Diabetes mellitus (DM) is a chronic condition affecting more than 451 million adults worldwide [1]. In Greece, type 2 diabetes mellitus (T2DM) has been estimated to affect 12% of the total adult population [2], demonstrating an increased prevalence as compared to older cross-sectional studies [3,4]. Depending on the type of the disease and the age of patients, treatment options include medication or insulin, self-management and lifestyle advice [5]. For patients with T2DM, adherence to non-insulin medication therapy is a challenging task, greatly dependent on the disease’s duration, with newly diagnosed patients being the less adherent ones [6,7]. In addition, 4% of patients with T2DM are “primary” non-adherents (i.e., they have never filled their prescriptions) [8]. Regarding insulin, only 62–64% of patients with T2DM comply with the prescribed dose [7], leaving approximately 1/3 of them without therapy. Among insulin-naïve patients, the rate of non-adherence is high: 4.5% are “primary” and an additional 25.5% “secondary” non-adherents (i.e., they fill their prescriptions in an inconsistent way) [7,9,10].

With regard to self-management, assessment of blood glucose concentrations is performed on average on 0.2–2.2 days each week, whereas foot care is implemented for approximately 2.2–4.3 days/weekly [11].

Following the lifestyle recommendations is another issue of concern among patients with DM [12]. Anxiety, depression and stress are associated with reduced compliance to the lifestyle-recommendations [13]. Long-term compliance to exercise goals can vary greatly, ranging between 10 to 80%, mainly due to lack of motivation and the fact that exercise is not monitored [10,14]. Concerning diet, patients often report disliking the foods listed in their meal plans [10] and tend to comply with the dietary recommendations for approximately 2.3–4.6 days every week [11]. Longer disease duration and the attainment of nutrition education appear to positively affect the rate of adherence [15].

Poor compliance is associated with poorer disease prognosis, an increased risk for developing comorbidities and complications [16], and a higher mortality ratio [10,17]. Understanding the level of adherence to the treatment recommendations is important to evaluate the quality of provided care, implement changes in the delivery of care and related education, and identify those less likely to adhere and improve patient outcomes. With this in mind, the present cross-sectional study aimed to evaluate adherence to the American Diabetes Association (ADA) nutrition recommendations among patients with T2DM.

## 2. Materials and Methods

### 2.1. Sample Recruitment and Inclusion and Exclusion Criteria

A total of 171 patients with a diagnosis of DM were recruited from the “Sismanoglio” General Hospital in Komotini, Northern Greece, during the year 2019 (25 June to 16 December). All out-patients visiting the clinic at the time of the study, fulfilling the inclusion criteria, were recruited. Pre-existing nutrition education was not a pre-requisite for participation. Given that the number of recruited patients with type 1 DM was low (*n* = 9), they were excluded from the analysis. Thus, the final sample consisted of 162 patients with T2DM.

Inclusion criteria were as follows: (1) adult patients, (2) diagnosis of T2DM, (3) ability to understand and articulate effortlessly in the Greek language, (4) willingness to participate, and (5) provision of informed consent. Exclusion criteria involved (1) patients not capable of consenting, (2) those unable to understand and communicate in Greek and (3) underage patients. The characteristics of the sample are provided in Table 1.

The study’s permission was granted by the “Sismanoglio” General Hospital Directorate (Approval ID: 457/20-June-2020), and all patients provided informed consent before participation. Data were handled according to the Helsinki Declaration for research on humans.

### 2.2. Adherence to the ADA Dietary Recommendations

According to the World Health Organization (WHO) [18,19], adherence for long-term treatment refers to “the extent to which a person’s behavior—taking medication, following a diet, and/or executing lifestyle changes, corresponds with agreed recommendations from a health care provider”. With this in mind, all ADA [20] nutrition recommendations issued in the same year of the present research (2019) were extracted by two researchers independently and included in a questionnaire with binary answers (yes/no). A total of 17 distinct recommendations were extracted from the guidelines [20] (Table 2). These were used to assess the degree of adherence to the guidelines. Two questions referring to insulin use were only addressed to those on insulin therapy (numbers 6 and 7—Table 2). No time restrictions were applied to participants regarding the completion of the questionnaire.

The percentage (%) of adherence to the ADA’s recommendations was calculated based on the sum of adherence (positive answers; “yes”) according to the 17 questions for patients on insulin therapy and based on the sum adherence of 15 questions for patients on therapy with oral antidiabetic agents (OAA) only.

For the purpose of the study, an experienced registered nutritionist dietitian (RDN) (S.K.) was present at the outpatient clinic for DM every day, throughout the study’s recruitment period. Participating patients were interviewed by the RDN on a one-on-one basis, and their reported adherence to the ADA recommendations was recorded on the questionnaire, on paper form, by the same RDN. No training was provided to the participants prior to participation.

For specific questions, participants were assisted with photographs of common food portion sizes and the micronutrient content of selected food groups, by the RDN. For instance, with regard to fiber, adequate intake was presented as daily portions of vegetables, fruit, or grains. Concerning sugar-sweetened beverages (SSBs), commercially available choices were presented to participants in order for them to report their intake. Alcohol portions were also presented in picture format, for participants to better understand and report their consumption. Commercially available sugar substitutes and dietary supplements were also presented, to achieve a more valid reporting.

### 2.3. Anthropometric and Other Characteristics of the Participants

The participants’ body weight and height were measured during morning hours with a digital scale (SECA 813, SECA Group, Hamburg, Germany) and a wall-mounted stadiometer (SECA 216, SECA Group, Hamburg, Germany). Body mass index (BMI) was calculated for each patient by dividing body weight (kg) by the stature (m) squared. Weight status was identified according to the World Health Organization thresholds for BMI [21].

Data concerning disease duration, comorbidities, latest HbA_1c_ assessment, education level, and medication therapy for DM were recorded for each participant. Given that Komotini has the greatest Muslim population in Greece, the religious denomination of participants was also recorded.

### 2.4. Statistical Analyses

Normality in distribution was assessed both visually and with the Shapiro–Wilk test. Normally distributed variables are presented as mean ± standard deviation (SD) and non-normally distributed as median with the respective interquartile range (IQR). Descriptive statistics for qualitative data are presented as frequencies (*n*) with their respective percentages in parentheses. Chi-square tests and the Mann–Whitney test were utilized to examine differences among qualitative and quantitative variables, respectively.

A univariable linear regression analysis was conducted in order to assess the impact on percentage adherence to the ADA’s recommendations total score (dependent variable) of the following study variables: age in years (continuous), sex (male vs. female), BMI in kg/m^2^ (continuous), educational level (primary vs. secondary or tertiary), religious denomination (Christian vs. Muslim), diabetes duration in years (continuous), HbA_1c_ in % (continuous), and therapy type (insulin vs. OAA only). A multivariable linear regression analysis was conducted, which included those independent variables selected by the augmented backward selection procedure as previously described [22]. Normality assumption and homoscedasticity were examined on the residuals, and multicollinearity was assessed by calculating tolerance and variance inflation factor (VIF). All analyses were carried out on the Statistical Software for Social Studies (SPSS) v. 25.0 (IBM SPSS Statistics, Armonk, NY, USA) and the SAS University Edition (SAS Institute, Cary, NC, USA). The significance level was set at α = 0.05.

## 3. Results

### 3.1. Adherence to the Dietary Recommendations

Total sample adherence to the ADA dietary recommendations [20] and adherence per participant group are presented in Table 2. The sample’s median percentage of adherence to the ADA dietary recommendations was suboptimal, reaching 41.2% (35.3%, 53.3%).

A mere 3.7% of the sample had received nutrition education by an RDN. The majority of participants reported adhering to the fiber (87.7%) and fish intake (78.4%) recommendations; most reported being aware of how to combat hypoglycemia (97.5%) and of the fact that supplements cannot improve glycemic control (79.6%). Only 9.9% of the patients followed an individualized diet plan to improve glycemic control, and 3.1% had specific energy targets set to reduce their body weight. Among those on insulin therapy, only 1.2% were competent to count their meals’ carbohydrate content, and 3.7% were receiving fixed insulin doses paired with a consistent carbohydrate intake. Recommendations regarding alcohol intake were only adhered to by 3.7% of the sample. The vast majority (87.7%) reported maintaining the pleasure of eating. Only 17.9% and 21.6% of the participants were able to control their blood lipid and blood pressure levels by diet alone, without pharmacotherapy.

Patients on OAA therapy only, exhibited increased adherence compared with those receiving insulin (medians: 46.7% vs. 38.2% respectively; *p* ≤ 0.001). In parallel, men were more compliant compared with women (medians: 46.7% vs. 41.2%, respectively; *p* ≤ 0.01) and younger patients compared with the older ones (medians: 46.9% vs. 41.2%, respectively; *p* ≤ 0.01).

### 3.2. Assessment of the Impact of Study Variables on Percentage of ADA Adherence Score

In a univariable assessment (Table 3), patients on OAA were associated with an increased percentage of ADA adherence score (β = 9.56, 95% CI = 6.45 to 12.63). A positive association was also observed among participants having attained education on the secondary or tertiary level (β = 6.28, 95% CI = 2.61 to 9.95). On the other hand, female sex and age were associated with a decreased percentage of ADA adherence score (β = −4.30, 95% CI = −7.56 to −1.04, and β = −0.22, 95% CI = −0.37 to −0.04). DM-related variables including diabetes duration (years) and HbA_1c_ (%) demonstrated a negative association with percentage of ADA adherence score (β = −0.51, 95% CI = −0.72 to −0.30, and β = 1.48, 95% CI = −2.50 to −0.46). Religious beliefs and BMI were not associated with the ADA adherence score (*p*-values > 0.05).

In the multivariable model (Table 3), patients on OAA therapy demonstrated increased adherence to the guidelines (by 8.36%), whereas adherence was decreased by 0.23% with every year of increasing age.

## 4. Discussion

Patients with T2DM exhibited low compliance to the ADA dietary recommendations for DM. Among those on OAA therapy only, men and younger patients reported a better adherence rate compared with patients receiving insulin, women and older patients. Based on the univariable analysis, age, BMI, DM duration, HbA_1c_, and Muslin denomination reduced compliance, whereas, having attained secondary or tertiary education and being on OAA therapy only, were associated with increased compliance. In the multivariable analysis, only OAA therapy and DM duration were associated with the rate of adherence to the recommendations.

In the present sample, only 3.7% of the participants had received nutrition education by an RDN. According to a systematic review and meta-analysis, the delivery of dietary education by RDNs consists of the most effective medium to improve patient outcomes [23]. Previous studies in Greece have revealed suboptimal nutrition knowledge among patients with DM [24,25,26], highlighting the need for an integrated approach to patient education. In parallel, randomized controlled trials (RCTs) have unanimously revealed improvements in glycemic control and disease prognosis following the implementation of nutrition education sessions [27,28]. In a study conducted in the USA [29] (Table 4), patients with T1DM and T2DM were educated on nutritional issues by an RDN and set individual goals based on the ADA/Morrison Health Care (MHC) recommendations. Post-intervention, 40% of participants attained the dietary goals excellently, 32.7% were adequately meeting their goals, and the remaining 27% adhered to the dietary goals fairly. A similar pattern was observed for exercise goals. After the intervention, an improvement was noted in the HbA_1c_ concentrations. In an RCT comparing adherence to the ADA dietary recommendations against a low-fat vegan diet, an improved overall adherence rate was noted in the latter compared with the former [30], indicating that many factors impeding adherence to the guidelines may exist. Interestingly, a mixed-methods study [31] failed to correlate DM knowledge to the rate of compliance with the guidelines (assessed by personal interviews). Several barriers were identified affecting the degree of compliance to the recommendations, including external stress, a conflict between advice and personal beliefs, lack of time and personal motivation, gaps in knowledge and living in an obesogenic environment [31]. Based on the latter observation, according to a recent meta-analysis [32], changing the dietary environment is more important for glycemic control compared with changing the dietary behavior; nevertheless, both are clinically effective for managing HbA_1c_ concentrations.

Apart from nutrition education, delivery of medical nutrition therapy (MNT) is important, as it involves the nutrition care process model, setting individual goals and guiding patients towards their attainment, close monitoring and evaluation. In the present sample, 9.9% of the patients were following an individualized diet plan to improve glycemia, whereas 3.1% had set specific energy targets and adhered to an MNT scheme aiming to reduce body weight. Individualized nutrition therapy carries more benefits for the patient than receiving dietary advice alone [33]. This is due to the low nutrition-related competencies and often inadequate education received by medical doctors in Greece and throughout the world [34,35] and the fact that RDNs are the most competent professionals for the delivery of MNT [36,37]. According to an econometric study [38], MNT is associated with a 9.5% reduction in the hospitalization of patients with DM, and a concurrent 23.5% decline in the use of physician services; thus, it should be considered as necessary instead of optimal, for all patients with DM [39].

Among participants in the present sample, complying with the alcohol recommendations proved to be a difficult task, as only 3.7% of them reported adhering to this goal. In contrast to the findings herein, in Northern California [40], most patients with DM complied with the ADA recommendations regarding alcohol intake. Alcohol intake is considered an indicator of poorer adherence to DM self-care behaviors [40]. In Greece, although alcohol use is not widespread, it holds a prominent position at celebrations and family meal tables. Thus, it might be difficult for patients with T2DM to control alcohol consumption in such habitual cases. Based on studies performed on humans and preclinical models, acute ethanol intake induces either a reduction, or a null effect on circulating glucose concentrations [41]. The first appears more likely to occur in fasted individuals, with only a small amount of evidence suggesting alcohol hypoglycemia in well-nourished patients [41,42,43,44]. Among patients with T2DM on different treatment regimes, it has been suggested that those on diet therapy do not experience hypoglycemia, whereas sulphonylurea-treated individuals might carry this risk [45].

Concerning the use of oral nutrient supplements (ONS) to improve glycemic control, most participants (79.6%) were aware that they are not efficient unless a deficiency is apparent, and subsequently, most patients herein abstained from their use. Nevertheless, 6.8% reported consuming cinnamon supplements, 1.9% received aloe vera ONS, 0.6% were reliant on curcumin to aid glycemic control, and 8% were consuming other supplements, with a special focus on poly-unsaturated fatty acids (PUFA). According to a recent scoping review [46], for many of the ONS postulated as efficient for DM care, a lack of scientific background is apparent. Thus, it is important to elaborate that these supplements often carry adverse effects and increase the cost of DM care, without offering any positive outcomes [47]. These issues can only be realized by educating patients on topics related to nutrition.

An individual’s diet is greatly dependent on an interplay of religious, familial, psychological, financial, and personal factors [48]. According to the ADA [49,50], structured interventions destined for patients with DM should be offered for diverse populations and audiences, integrating both culture and religion. In particular, religion is an integral part of the culture, often directing food habits through Lent, fasting or beliefs related to certain foods [48]. Concerning the effect of the religious denomination on the rate of adherence to the dietary guidelines, no differences were observed in the total compliance score herein, apart from a greater proportion of patients with Christian denomination following specific energy targets for bodyweight reduction and reporting using sugar substitutes to a greater extend. Although having a Muslim denomination was associated with a decreased adherence score in the univariable analysis, this effect failed to remain significant when all other factors were considered. This fact indicates that in Komotini’s multicultural community, healthcare equity does not allow for disparities based on religion, offering an individually adapted DM-care model.

Based on the multivariable model, each yearly increment in the age reduced total adherence score by −0.23%, whereas those on OAA therapy only had an increased dietary compliance score by 8.36%. These findings agree with previous research on patients with T2DM, suggesting that the use of insulin is associated with a decreased adherence to the therapy [51]. With regard to age, although a study in Rio de Janeiro revealed a decreased compliance rate among older patients [52], conflicting evidence exists in the literature concerning this issue. Concerning diet, studies have revealed an improved adherence rate with a more recent DM diagnosis (and subsequently, younger age) [53]; concerning medication, increasing age appears to ameliorate adherence [6,7,54,55].

The results also indicated a reduced adherence rate among women participants as compared to their male counterparts. Previous research on Greek patients with DM has also revealed a reduced nutritional knowledge and self-management practices among women with T2DM inhabiting the city of Agrinio, in Central Greece [24,26]. Apart from the fact that older women inhabiting mainland Greece are likely to have received less education in general, women are also responsible for preparing the meals for all the family. This increased preoccupation with food, including frequent tasting of the prepared dishes, is likely a factor reducing adherence to the dietary recommendations among women [25]. In parallel, according to Fitzgerald [56], gender differences are also apparent in diabetes attitudes. On the other hand, according to a study in Singapore [57], lower adherence was demonstrated in men with DM as compared to women. Therefore, the exact extent of the effect of gender on DM adherence remains unclear.

Table 4 summarizes available evidence assessing adherence to the dietary recommendations among patients with DM. In Canada [58], patients with T2DM exceeded daily sodium and saturated fatty acid (SFA) recommendations issued by the Canadian Diabetes Association (CDA). Cured meats, fast foods and snack foods were all major contributors to the intake of sodium and saturated fat [58]. In Pakistan [59], 36.5% of patients with DM complied with the ADA dietary recommendations. In further detail, greater adherence was noted among those feeling comfortable with their diet plan, and those aware of which diet was harmful to them. In Italy, a great proportion of patients with T2DM adhered to the recommendations for protein and added sugars, but the respective percentage of those meeting SFA and fiber goals was lower [60]. A similar pattern was noted in Thailand [61], with the majority of patients with T2DM failing to meet local SFA and fiber recommendations, issued based on the ADA respective guidelines. In Finland [62], a mere 28% of patients with T1DM with nephropathy restricted their SFA intake according to the recommended levels of less than 10% of the total energy intake based on the Finish Diabetes Association (FDA) guidelines. In parallel, 4% of the participants met the recommended fiber intake values, and 25% exceeded the suggested goal regarding sugar intake [62]. Nevertheless, more than 50% of the participants perceived themselves as adherers to the dietary recommendations either “most of the time”, or “always” [62]. 

Concerning adolescents with T1DM [64], according to a study from Norway, apart from the intake of fat and fiber, which were over- and under-consumed, respectively, all intakes of remaining macronutrients were adequate according to the recommendations of the relevant International Society for Pediatric and Adolescent Diabetes (ISPAD). Improved dietary intakes were also noted among adolescents with better glycemic control.

The present study indicates that MNT and nutrition education are often neglected areas of DM care in Greece. Most hospitals fail to employ an adequate number of RDNs, and, thus, nutritional education and care are often offered in private practice to those patients who have the financial means to address relevant out-of-pocket expenses. On the other hand, multidisciplinary teams are required for the management of all conditions/diseases, and as far as DM is concerned, systematic reviews and meta-analyses have highlighted the importance of including RDNs in routine DM care for improved patient outcomes [65].

The limitations of this study include the assessment of adherence to the ADA guidelines instead of relevant recommendations from a Greek society/authority. Furthermore, the cross-sectional design does not allow for causal associations. In parallel, using a relatively small sample from one hospital only might not allow for extrapolation of the findings (external validity).

## 5. Conclusions

According to the WHO, among patients with chronic disease, the rate of adherence to lifestyle recommendations is lower as compared to medication ones [19]. A salient explanation for this phenomenon might be that medication therapy does not require particular effort from the part of the patient, whereas on the other hand, lifestyle treatment is greatly dependent on the stage of motivational readiness [66,67]. The present cross-sectional study demonstrated a low reported adherence rate to the dietary recommendations for DM among patients with T2DM, indicating the need for improved nutrition education and diet care.

## Figures and Tables

**Table 1:** Patient characteristics (*n* and %, or mean ± SD) (*n* = 162).

Men/Women (*n*, %)	67 (41.4%)/95 (58.6%)
Age (years)	64.7 ± 10.6
Bodyweight (kg)	86.7 ± 18.0
Height (cm)	164 ± 8
BMI (kg/m^2^)	32.4 ± 6.6
Weight status (normoweight/overweight/obese) (*n*, %)	13 (8%)/60 (37%)/89 (55%)
Educational level (primary/secondary/tertiary) (*n*, %)	122 (75.3%)/24 (14.8%)/16 (9.9%)
Religion (Christian/Muslim)	101 (62.3%)/61 (37.7%)
Diabetes diagnosis (years)	10.7 ± 7.3
HbA_1c_ (%)	7.1 ± 1.6
Therapy (insulin/pharmacotherapy/both)	22 (13.6%)/104 (64.4%)/36 (22.2%)
SBP (mm Hg)	129.1 ± 18.3
DBP (mm Hg)	80.3 ± 7.6
Triglycerides (mg/dL)	157.8 ± 98.5
HDL (mg/dL)	46.5 ± 11.9
LDL (mg/dL)	95.0 ± 31.4
TSH (mU/L)	2.2 ± 1.0

BMI, body mass index; DBP, diastolic blood pressure; HbA_1c_, glycosylated hemoglobin; HDL, high-density lipoprotein; LDL, low-density lipoprotein; SBP, systolic blood pressure; SD, standard deviation; TSH, thyroid-stimulating hormone. Missing values: LDL (*n* = 41), HDL (*n* = 37), Triglycerides (*n* = 70), SBP (*n* = 24), DBP (*n* = 24), and TSH (*n* = 137).

**Table 2:** Patient adherence to individual nutrition recommendations suggested by the American Diabetes Association (ADA) (*n* = 162) (*n*, % or median, interquartile range (IQR)).

	Individual Nutrition Recommendations:	Therapy		Sex		Religious Denomination		Age Group		Total (*n* = 162)
		On OAA Only (*n* = 104)	On Insulin ^$^ (*n* = 58)	Men (*n* = 67)	Women (*n* = 95)	Christian (*n* = 101)	Muslim (*n* = 61)	<60 Years Old (*n* = 50)	≥60 Years Old (*n* = 112)	
1	Follow an individualized diet plan based on personal needs to improve glycemia	12 (11.5%)	4 (6.9%)	9 (13.4%)	7 (7.4%)	12 (11.9%)	4 (6.6%)	8 (16%)	8 (7.1%)	16 (9.9%)
2	Consumption of nutrient-dense foods in appropriate portion sizes in order to improve overall health	33 (56.9%)	55 (52.9%)	35 (52.2%)	53 (55.8%)	59 (58.4%)	29 (47.5%)	27 (54%)	61 (54.5%)	88 (54.3%)
3	Follow an intensive lifestyle intervention program including individualized goals and/or to individualized MNT (for people with prediabetes or overweight/obesity)	37 (35.6%) *	11 (19%)	22 (32.8%)	26 (27.4%)	36 (35.6%) *	12 (19.7%)	19 (38%)	29 (25.9%)	48 (29.6%)
4	Consumption of a specific energy target each day based on the individualized body weight goals and needs	4 (3.8%)	1 (1.7%)	3 (4.5%)	2 (2.1%)	5 (5%)	0 (0%)	3 (6%)	2 (1.8%)	5 (3.1%)
5	Consumption of adequate dietary fiber (preferably through food (vegetables, pulses (beans, peas and lentils), fruits, and whole intact grains)	96 (92.3%)	46 (79.3%)	62 (92.5%)	80 (84.2%)	91 (90.1%)	51 (83.6%)	43 (86%)	99 (88.4%)	142 (87.7%)
6	Able to count carbohydrate content of foods to calculate appropriate insulin dose	0 (0%) ^∂^	2 (3.4%)	2 (3%) *	0 (0%)	1 (1%)	1 (1.6%)	2 (4%) **	0 (0%)	2 (1.2%)
7	Use fixed daily insulin doses, consistent carbohydrate intake with respect to time and amount	0 (0%) ^∂^	6 (10.3%)	5 (7.5%)	1 (1.1%)	4 (4%)	2 (3.3%)	5 (10%)	1 (0.9%)	6 (3.7%)
8	Consumption of SSBs (including juice with sugar, energy drinks and soft-drinks) ^#^	35 (33.7%)	22 (37.9%)	22 (32.8%)	35 (36.8%)	30 (29.7%)	27 (44.3%)	22 (44%)	35 (31.3%)	57 (35.2%)
9	In the case of hypoglycemic episodes:									
	a. Consumption of carbohydrate in the form of juice or sugar	101 (97.1%)	57 (98.3%)	63 (94%) *	95 (100%)	99 (98%)	59 (96.7%)	49 (89%)	109 (97.3%)	258 (97.5%)
	b. Consumption of carbohydrate food with high protein content (i.e., bread or cereals)	3 (2.9%)	1 (1.7%)	4 (6%) *	0 (0%)	2 (2%)	2 (3.3%)	5 (10%)	1 (0.9%)	4 (2.5%)
10	Consumption of one serving of fish (particularly fatty fish), at least twice/weekly	83 (79.8%)	44 (75.9%)	53 (79.1%)	74 (77.9%)	84 (83.2%)	43 (70.5%)	36 (72%)	91 (81.3%)	127 (78.4%)
11	Routine use of the following supplements for improving glycemia:									
	Vitamin D ^‡^	3 (2.9%)	2 (3.4%)	0 (0%)	5 (5.3%)	4 (4%)	1 (1.6%)	1 (2%)	4 (3.6%)	5 (3.1%)
	Chromium ^‡^	0 (0%)	0 (0%)	0 (0%)	0 (0%)	0 (0%)	0 (0%)	0 (0%)	0 (0%)	0 (0%)
	Curcumin ^‡^	1 (1%)	0 (0%)	1 (1.5%)	0 (0%)	0 (0%)	1 (1.6%)	1 (2%)	4 (3.6%)	1 (0.6%)
	Cinnamon ^‡^	9 (8.7%)	2 (3.4%)	3 (4.5%)	8 (8.4%)	9 (8.9%)	2 (3.3%)	6 (12%)	5 (4.5%)	11 (6.8%)
	Aloe vera ^‡^	3 (2.9%)	0 (0%)	2 (3%)	1 (1.1%)	3 (3%)	0 (0%)	1 (2%)	2 (1.8%)	3 (1.9%)
	Any supplement ^ƒ^	6 (5.8%)	7 (12.1%)	6 (9%)	7 (7.4%)	12 (11.9%)	1 (1.6%)	4 (8%)	9 (8%)	13 (8%)
	No supplement	82 (78.8%)	47 (81%)	55 (82.1%)	74 (77.9%)	73 (72.3%)	56 (91.8%)	37 (74%) **	92 (82.1%)	129 (79.6%)
12	When alcohol is consumed, this is done in moderation (≤1 drink/day for women and ≤2 drinks/day for men)	4 (3.8%)	2 (3.4%)	6 (9%) **	0 (0%)	4 (4%)	2 (3.3%)	1 (2%)	5 (4.5%)	6 (3.7%)
13	Use of sugar substitutes (i.e., stevia, saccharin, acesulfame-K, aspartame, sucralose, etc.) ^†^	18 (17.3%) *	20 (34.5%)	20 (29.9%)	18 (18.9%)	29 (28.7%) *	9 (14.8%)	12 (24%)	26 (23.2%)	38 (23.5%)
14	Maintenance of the pleasure of eating	96 (92.3%)	46 (79.3%)	62 (92.5%)	80 (84.2%)	91 (90.1%)	51 (83.6%)	45 (90%)	97 (86.6%)	142 (87.7%)
15	Controlling blood lipid levels through diet	22 (21.2%)	7 (12.1%)	17 (25.4%)	12 (12.6%)	21 (20.8%)	8 (13.1%)	14 (28%)	15 (13.4%)	29 (17.9%)
16	Controlling arterial blood pressure with diet	27 (26%)	8 (13.8%)	21 (31.3%) *	14 (14.7%)	26 (25.7%)	9 (14.8%)	20 (40%) ***	15 (13.4%)	35 (21.6%)
17	Received comprehensive nutrition education provided by a RDN, preferably one with DM knowledge and experience	4 (3.8%)	2 (3.4%)	4 (6%)	2 (2.1%)	6 (5.9%)	0 (0%)	3 (6%)	3 (2.7%)	6 (3.7%)
	Adherence to the recommendations (% of correct answers) ^∝^^§^	46.7 (40, 53.3) ***	38.2 (29.4, 41.2)	46.7 (40, 53.3) **	41.2 (35.3, 52.9)	46.7 (40, 53.3)	41.2 (35.3, 47.1)	46.9 (40, 53.3) **	41.2 (35.3, 50.2)	41.2 (35.3, 53.3)

ADA, American Diabetes Association; CVD, cardiovascular; DM, diabetes mellitus; IQR, interquartile range; MNT, medical nutrition therapy; OAA, oral antidiabetic agents; RDN, registered dietitian -nutritionist; SSBs, sugar-sweetened beverages. ^$^ Some were additionally receiving OAA; ^#^ should be avoided; ^†^ do not appear to reduce long-term CVD risk or body weight; ^‡^ not recommended; ^ƒ^ not recommended, unless a deficiency is present; ^∂^ For those on OAA, these two questions were omitted from the adherence score; ^∝^ Based on 17 questions for those on insulin and 15 questions for those on OAA therapy only; * Significantly different compared to the opposite group (on insulin therapy/women/Muslim denomination/age ≥ 60 years old) as follows *** *p* ≤ 0.001, ** *p* ≤ 0.01, * *p* ≤ 0.05; ^§^ Tested with the Mann–Whitney test.

**Table 3:** Univariable and multivariable linear regression assessment of the impact of study variables on the percentage of the total adherence score.

Independent Variable	Univariable		Multivariable ^†^	
	β Coef (95% CI)	Significance	β Coef (95% CI)	*p*-Value
Age	−0.22 (−0.37 to−0.04)	0.006	−0.13 (−0.29 to 0.03)	0.115
Female	−4.30 (−7.56 to −1.04)	0.010	−2.26 (−5.15 to 0.63)	0.125
BMI (kg/m^2^)	−0.10 (−0.35 to 0.15)	0.450	-	
Secondary/tertiary education	6.28 (2.61 to 9.95)	0.001	3.84 (−0.003 to 7.69)	0.050
Muslim denomination	−3.01 (−6.36 to 0.34)	0.077	−2.31 (−5.58 to 0.96)	0.165
Diabetes duration (years)	−0.51 (−0.72 to −0.30)	<0.001	−0.23 (−0.46 to −0.01)	0.042
HbA_1c_ (%)	−1.48 (−2.50 to −0.46)	0.005	-	
OAA therapy only	9.56 (6.48 to 12.63)	<0.001	8.36 (5.24 to 11.74)	<0.001

β Coef, linear regression coefficients; BMI, body mass index; CI, confidence intervals; HbA_1c_, glycosylated hemoglobin; OAA, oral antidiabetic agents. ^†^ included variables selected by the augmented backward selection procedure [22] with adjusted R^2^: 0.306.

**Table 4:** Cross-sectional studies assessing adherence to the dietary guidelines among patients with diabetes.

First Author	Origin	Recruitment	Patient Characteristics			RR (%)	Guidelines Evaluated	Tools	Results
			DM Type	Age (Years)	N				
Ahmed [40]	US	Kaiser Permanente Northern California Diabetes Registry, and patients from the pharmacy, laboratory, outpatient clinics, ER and hospitalization	T1DM and T2DM	58.1 ± 13 *	77,722 (Alcohol drinkers: 38,564)	83	ADA	Survey by mail, computer-assisted telephone interview, HbA_1c_ and further DM-related tests	Among current drinkers, 92% of men (2 drinks/day) and 94% of women (1 drink/day) adhered to the guidelines. Alcohol consumption was inversely associated with HbA_1c_.
Ahola [62]	FI	Patients of the Finnish Diabetic Nephropathy Study “FinnDiane”	T1DM	Men: 51 (40–60) ^†^ Women: 47 (37–54) ^†^	817	63	FDA	FFQ, self-reported compliance with guidelines, 3-day food records concerning food intake, PA, Ins dose, and BG levels	Only 28% of participants restricted SFA to the recommendation. Almost 1/4 had higher than recommended sucrose intake. Fiber goals were met by 4% of participants.
Gillani [59]	PK	Patients from hospitals in Multan, Bahawalpur, and Rahim Yar Khan	T1DM and T2DM	48.8 ± 14.6 *	398	44.3	ADA	3-day recalls were used to assess dietary history and diet compliance	Diet compliance was 36.5%. Patients feeling comfortable with their diet plan and those aware of which diet was harmful to them were more compliant.
Krige [63]	ZA	Women were diagnosed during pregnancy from two hospitals	GDM/IGT	32.2 ± 5.3 *	239	--	SEMDSA	Interview administered picture-quantified FFQ and beliefs concerning food consumption	The average protein constituted 14.7% of TEI (goal 20%), with 93.5% of patients consuming protein below the cut-off point. The average carbohydrate intake was 53% (goal 40%), with 92.2% exceeding the goal. Mean fat intake was 33% of TEI (goal 40%), and 80.4% of patients were below the goal. Overall, patients demonstrated moderate adherence.
Nedra [29]	US	Hospital outpatients, a freestanding and an endocrinology clinic	T1DM and T2DM	56 ± 16.7 *	102	NR	ADA and MHC	Data from chart notes and phone calls for patients’ self-rating of their DM knowledge, before and after nutrition education by an RDN	Approximately 40% of participants attained their goals excellently, 32.7% rated meeting their goals as good, and the remaining 27% considered their adherence as fair.
Øverby [64]	NO	Norwegian Childhood Diabetes and Quality project	T1DM	11.3 ± 3.4 *	550	34	DNSG	4-day food records, FFQ, questionnaire on parental education, diabetes examinations	Apart from the intake of fat and fiber, which were higher and lower respectively than recommended, all intakes of macronutrients were adequate according to current recommendations.
Raj [58]	CA	Through advertising	T2DM	61.2 ± 10.4 *	80	80	CDA	PDAQ, 3-day food records, anthropometry and DM examinations	Reported mean daily intakes of Na and SFA exceeded the recommendations. Cured meats, fast foods and snack foods were all major contributors to intake of Na and SFA. SFA, Na intakes and total PDAQ scores did not correlate with HbA_1c_ concentration.
Thewjitcharoen [61]	YH	Outpatients of the DM clinics from the Theptarin and Ramathibodi hospitals	T2DM	57.4 (25–85) ^†^	304	NR	Thailand guidelines based on the ADA	3- or 7-day food records, nutritional knowledge and a dietary self-care behavior questionnaire	Low adherence to the local guidelines was observed. The recommended intake of SFA was met by only 32.7% of free sugars by 11.8% and fiber by 1.6%.
Vitale [60]	IT	57 centers throughout Italy	T2DM	62.1 ± 6.5 *	2568	NR	DNSG and SID	EPIC FFQ, and specific software to convert dietary data to average daily amounts of foods	Adherence to the recommendations was high for the consumption of protein (77.8%) and added sugars (97.3%), while adherence for the intake of recommended quantities of SFA (17.9%) and fiber (6.9%) was lower.

ADA, American Diabetes Association; BG, blood glucose; CDA, Canadian Diabetes Association; DGA, Dietary Guidelines for Americans; DNSG, Diabetes and Nutrition Study Group; DM, diabetes mellitus; EPIC, European Prospective Investigation into Cancer and Nutrition; ER, emergency room; FDA, Finish Diabetes Association; FFQ, food frequency questionnaire; GDM, gestational diabetes mellitus; HbA_1c_, glycosylated hemoglobin; HEI, Healthy Eating Index; IGT, impaired glucose tolerance; Ins, insulin; ISPAD, International Society for Pediatric and Adolescent Diabetes; MDG, Malaysian Dietary Guidelines; MHC, Morrison Health Care; Na, sodium; NCDQ, Norwegian Childhood Diabetes and Quality; NR, not reported; PA, physical activity; PDAQ, Perceived Dietary Adherence Questionnaire; RDN, registered nutritionist dietitian; RR, response rate; SD, standard deviation; SEMDSA, Society for Endocrinology Metabolism an Diabetes of South Africa; SID, Italian Diabetes Society; SFA, saturated fatty acids; T1DM, type 1 diabetes mellitus; T2DM, type 2 diabetes mellitus; TEI, total energy intake; USDA, United States Department of Agriculture. ^†^ Mean (range); * Mean ± SD.
